# Emmet’s Operation

**Published:** 1879-07

**Authors:** E. C. Dudley

**Affiliations:** Chicago, Ill.


### Domestic Correspondence.

---

ARTICLE XIII.

CHICAGO, Ill., June 18, 1879.

*To the Editors of* THE CHICAGO MEDICAL JOURNAL AND EXAMINER,

*Gentlemen :*—That I may reply explicitly to the communication of Dr. Robert Barnes, which appeared in your last number, permit me to introduce his letter in full:

LONDON, 15 HARLEY STREET, W., May 3, 1879.

*To the Editor of* THE CHICAGO MEDICAL JOURNAL AND EXAMINER,

*Sir:*—My attention has been called to an article in your issue of March last on Emmet's Operation," by Dr. E. C. Dudley, in which occurs the following passage: " Either from absolute ignorance, or from profound prejudice, or from a carelessness which in the law would amount to malice, the most exhaustive gynæcological works in England (Barnes), France (Leblond), and Germany (Hegar, Kaltenbach, Schrœder) are absolutely silent."

" Big words!" It is not my affair to inquire how far they may be justly applied to Leblond, Hegar, Kaltenbach and Schrœder. As to me they possibly might be applied, *if the allegation were only true* that I had been " absolutely silent " about " Emmet's operation." But as a matter of fact I have mentioned it, and with the unqualified respect which I entertain for one of the most able and illustrious of my American friends. At page 873, 2d edtiion of my " Diseases of Women," 1878, I give a compendious summary of Emmet's operation, quoting the source (*American Journal of Obstetrics*, 1874), and I conclude with the following appreciation: " I can confirm the accuracy of Emmet's views. I have performed his operation with satisfactory results."

So if there is " absolute ignorance " it is not mine: if there is " profound prejudice," it is not mine; and if there is " carelessness amounting to malice," it is not mine. It is possible that Dr. Dudley is misled by copying a similar impeachment made against me by Dr. Paul Mundé, in the *American Journal of Obstetrics*, instead of reading my book for himself. Dr. Mundé has since apologized to me for his error. Dr. Dudley will no doubt do the same. As I am entitled to damages I claim the right to fix the penalty. I condemn him to read my book, and caution him never again to accept statements at second hand. I am, sir, yours sincerely,
ROBERT BARNES.

The following is a quotation in full of all that is contained in the last edition of Dr. Barnes' book on the subject of laceration of the cervix:

"Emmet calls attention to the frequency of the laceration of the cervix, and to the serious distress they entail. (*Am. Jour. of Obst.*, 1874). They are most frequent, he says, in the median line, and more frequent in the anterior than in the posterior lip. If in the median line and confined to the cervix, they generally heal rapidly. But when the laceration extends through the vesico-vaginal septum a fistula may result; and this part of the rent may not heal. Lacerations through the posterior lip also heal rapidly; but sometimes when they extend far back, inflammation may arise, and *cause* an intractable form of retroversion. A cicatricial band is left, which must be removed before relief is obtained. But it is the lateral lacerations with which we are chiefly concerned in practice. Whenever the laceration has extended to the vaginal junction or beyond, there exists a tendency for the tissue to roll out from within the cervical canal. Hypertrophy and cystic degeneration of the lips ensue; involution is arrested, partial obliquity of the uterus is produced. On getting about, the woman is soon the subject of great distress; leucorrhœa, menorrhagia, dysmenorrhœa, difficulty in walking impel her to consult her physician. The attendant abrasion, glandular inflammation, hypertrophy, leucorrhœa are readily recognized, and many such cases are treated for ulceration ineffectually for months. Emmet has successfully treated more than two hundred such cases by paring the edges of the fissure, and uniting them by silver sutures, so restoring the cervix to its normal shape. I can confirm the accuracy of Emmet's views. I have performed his operation with satisfactory results."

It will be observed that Dr. Barnes devotes about one-half of a page to one of the most important, if not the most important, subject treated in his book, and about an entire page to making good his defense against the charge of absolute silence.

When the last edition appeared, I examined it carefully with special reference to laceration of the cervix, but failed to find anything. I am, therefore, unable to profit by the explanation charitably offered by Dr. Barnes, that I was misled by the similar error of Dr. Mundé, nor is it certain that Dr. Barnes does himself justice in presenting his book to be read as a penance. I read it with pleasure.

Had I been alone in failing to discover that Dr. Barnes "mentioned" this subject, such a failure might perhaps be attributed to gross carelessness, but Dr. Barnes says that the editor of the *American Journal of Obstetrics* did the same thing. Others might be mentioned, of whom one enjoys a degree of eminence not unequal to that of Dr. Emmet himself. The following is from Emmet's book.

"As soon as the practitioner becomes able to recognize this lesion under its different forms, he will be surprised to find a new explanation of all his cases of elongated or hypertrophied cervix, as well as for those of ulceration. * * * * * let any one once master the diagnosis and he will not fail to recognize the protean nature of lacerations, * * * * he will never have occasion afterwards to amputate the cervix, or any portion of it, except for malignant diseases."

Dr. Barnes, on subinvolution, hypertrophy of the cervix, cystic degeneration, "ulceration," and granular erosion, utterly ignores laceration as a cause, although it is generally the cause. His plates also (figures, 101, 103, 117, 119, 120, 121, 122) used to illustrate the so-called hypertrophied cervix, are excellent illustrations of laceration.

It is unfortunate that such plates are employed for such a purpose. In the treatment of this supposed hypertrophy and elongation amputation is sometimes advised, although for several years it has been demonstrated in the Woman's Hospital at New York and elsewhere, that these conditions may be entirely removed by suture. In the index may be found the words, *Cervix Uteri, injures to, in labor*, page 426. Here follow five pages in which laceration of the cervix is not "mentioned;" but two cuts are introduced, which whould admirably serve to illustrate the subject in a future edition.

Having failed to discover any mention of laceration, under diseases of the cervix and fundus uteri, and under injury to the cervix during labor, I supposed the author to have been "absolutely silent;" but had it occurred to me to look carefully under diseases of the vagina (which I confess it did not) I should have found a short paragraph, an abstract of one of Emmet's Memoirs, already quoted above, wedged in between sloughing of the vagina on one side and vesico-vaginal fistula on the other. It is difficult to imagine that any one not familiar with the subject would gain even a suspicion of its importance by reading this paragraph. I now substitute for "*absolutely silent*," *practically silent.*

It is a principle of the law, that when one of two parties engaged in a dispute must suffer, that one shall suffer who by his voluntary act has made it possible for the dispute to arise. Since Dr. Barnes, by his voluntary act, has rendered possible this dispute, I am clearly the aggrieved party and he is the culprit. I desire to fix the penalty.

Dr. Barnes will overlook my oversight and that of Dr. Mundé, and publish an improved edition of his very valuable work immediately.

E. C. Dudley.

---

Cuprum Ammoniatum in Neuralgia of the Fifth (*Bulletin de l'Académie de Médecine.*) (Seance of 1st April, 1879. By Dr. Féréol.) Dr. Féréol has obtained marked and sometimes instantaneous relief from the exhibition of cuprum ammoniatum, in obstinate cases of neuralgia of the fifth pair of nerves. He does not claim to have found an infallible remedy, but modestly asks for it a trial in this troublesome affection. In one or two of the cases the patients who were relieved had, previous to its administration been deprived of sleep for weeks. The commencing dose should vary from Gr. 0.10 to 0.15 a day, gradually increased to Gr. 0.30 or even 0.50, carefully watching the susceptibility of each individual. It is best administered in pills or capsules, and the daily amount above indicated should be divided into eight or ten parts, to be taken at intervals preferably with food. It is important to continue the treatment for twelve or fifteen days after the cessation of pain.

Amyl Nitrate a Cardiac Stimulant.—There is an accumulation of evidence that this drug is a prompt and valuable stimulant. Where a rapid action is desired, it has no equal. Even when inhaled in two-gram and four-gram doses, it has never done harm. Physicians should overcome their fear in this regard.

